# Supplementary figures and images for: Incidence of Prostate Cancer in Inflammatory Bowel Disease: A Meta-Analysis
Source: Medicina (Kaunas). 2020 Jun 11;56(6):285. doi: 10.3390/medicina56060285 (PMC7353864; doi:10.3390/medicina56060285)

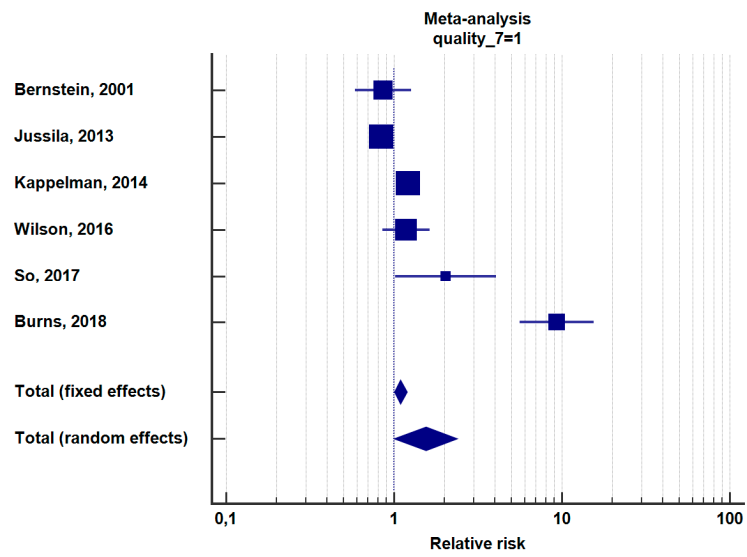

a)

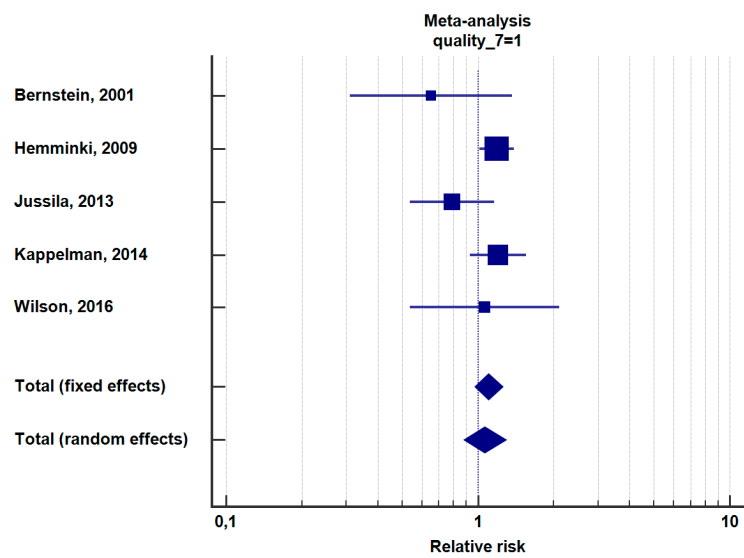

b)

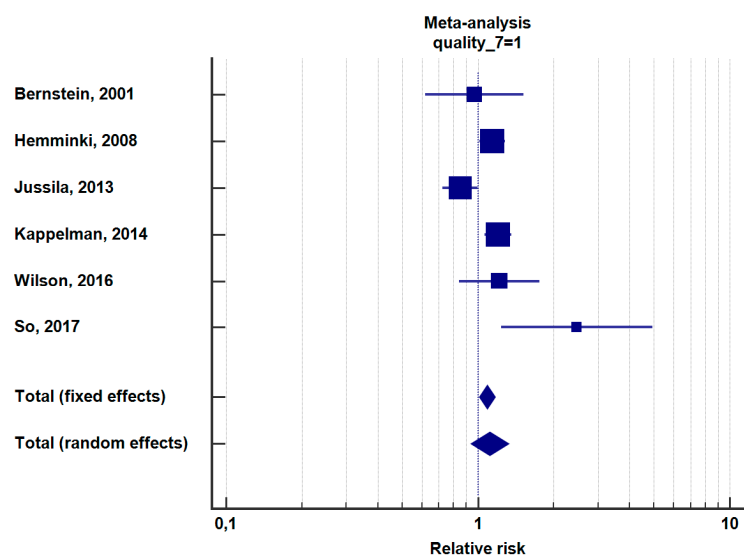

c)

Figure S1: forest plot quality subanalysis for RR of PC in a) IBD, b) CD and c) UC

Supplement: Supplementary file 1 [file medicina-56-00285-s001.pdf]
